# Supplementary material for: Organic matter degradation by oceanic fungi differs between polar and non-polar waters
Source: Nat Commun. 2025 Aug 15;16:7589. doi: 10.1038/s41467-025-63047-4 (PMC12356891; doi:10.1038/s41467-025-63047-4)
Supplement: Supplementary file 1 — Supplementary Information [file 41467_2025_63047_MOESM1_ESM.pdf]

Supplementary Materials

Organic matter degradation by oceanic fungi differs between polar and non-polar waters

Kangli Guo[1]*, Zihao Zhao[1], Eva Breyer[1,2], Federico Baltar[1,2]*

[1] Fungal and Biogeochemical Oceanography Group, Department of Functional and Evolutionary Ecology, University of Vienna, 1030 Vienna, Austria

[2] Fungal and Biogeochemical Oceanography Group, College of Oceanography and Ecological Science, Shanghai Ocean University, Nanhui New City, 201306 Shanghai, China

*Correspondence to: kangli.guo@univie.ac.at; fbaltar@shou.edu.cn

This file includes:

Supplementary Figures S1 to S11

Other Supplementary Material for this manuscript includes the following:

Supplementary Data 1-7

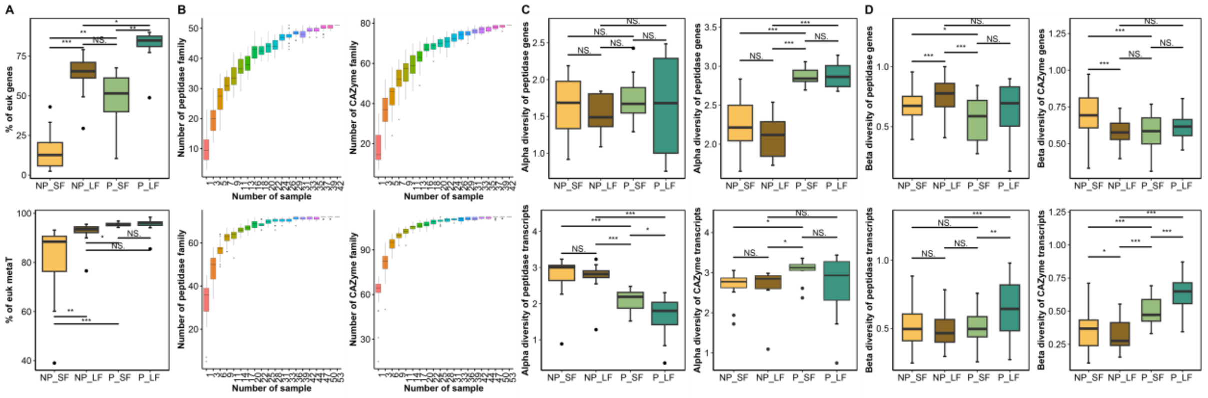

Fig. S1 Percentage distribution of eukaryotic sequences. (**A**) Rarefaction curve of detected genes and transcripts encoding peptidase and CAZyme (**B**) and Shannon index of total peptidase (in the left panel of Figure C) and CAZyme (in the right panel of Figure C) genes and transcripts (**C**) The β-diversity of total peptidase (in the left panel of Figure D) and CAZyme (in the right panel of Figure D) genes and transcripts (**D**) Statistics are based on the two-sided t-test. NS., not significant, * p<0.05, ** p<0.01, **p<0.001.

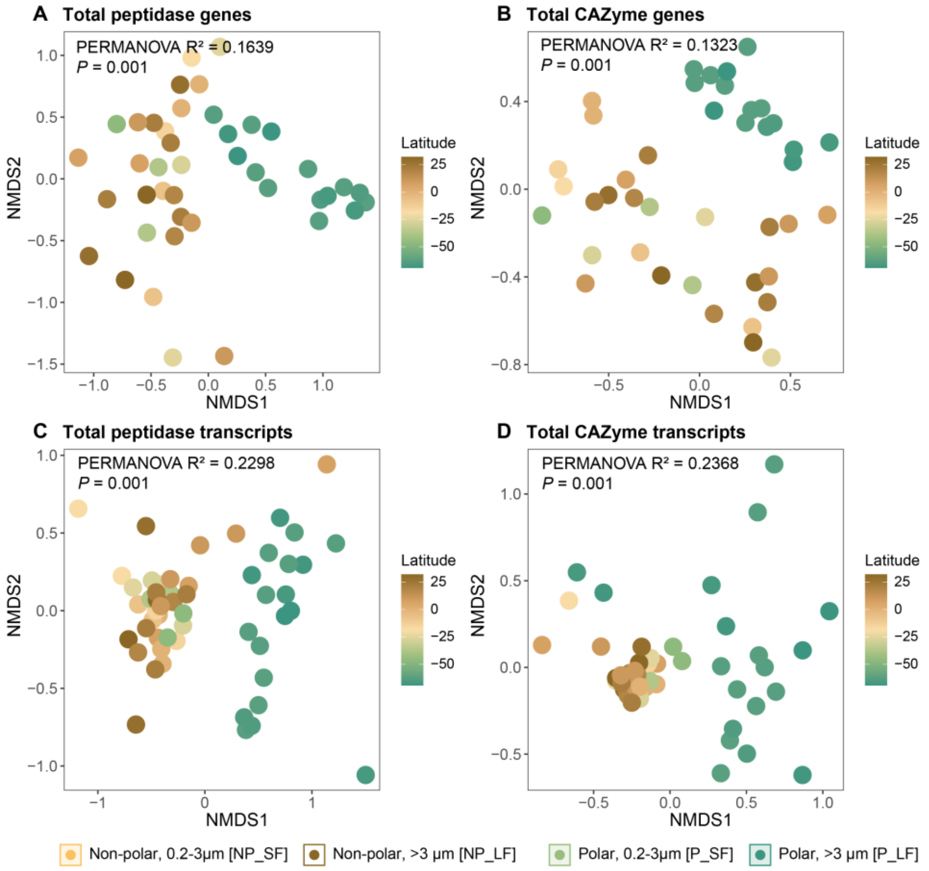

Fig. S2 High-resolution spatial distribution of genes and transcripts encoding fungal peptidases and CAZymes along a latitudinal gradient. Non-metric Multidimensional Scaling (NMDS) based on the relative abundance of genes (**A, B**) and transcripts (**C, D**) of total fungal peptidases (**A, C**) and CAZymes (**B, D**). Different colors are used to represent the samples collected along a latitudinal gradient. Pearson correlation coefficients (two-sided) were calculated.

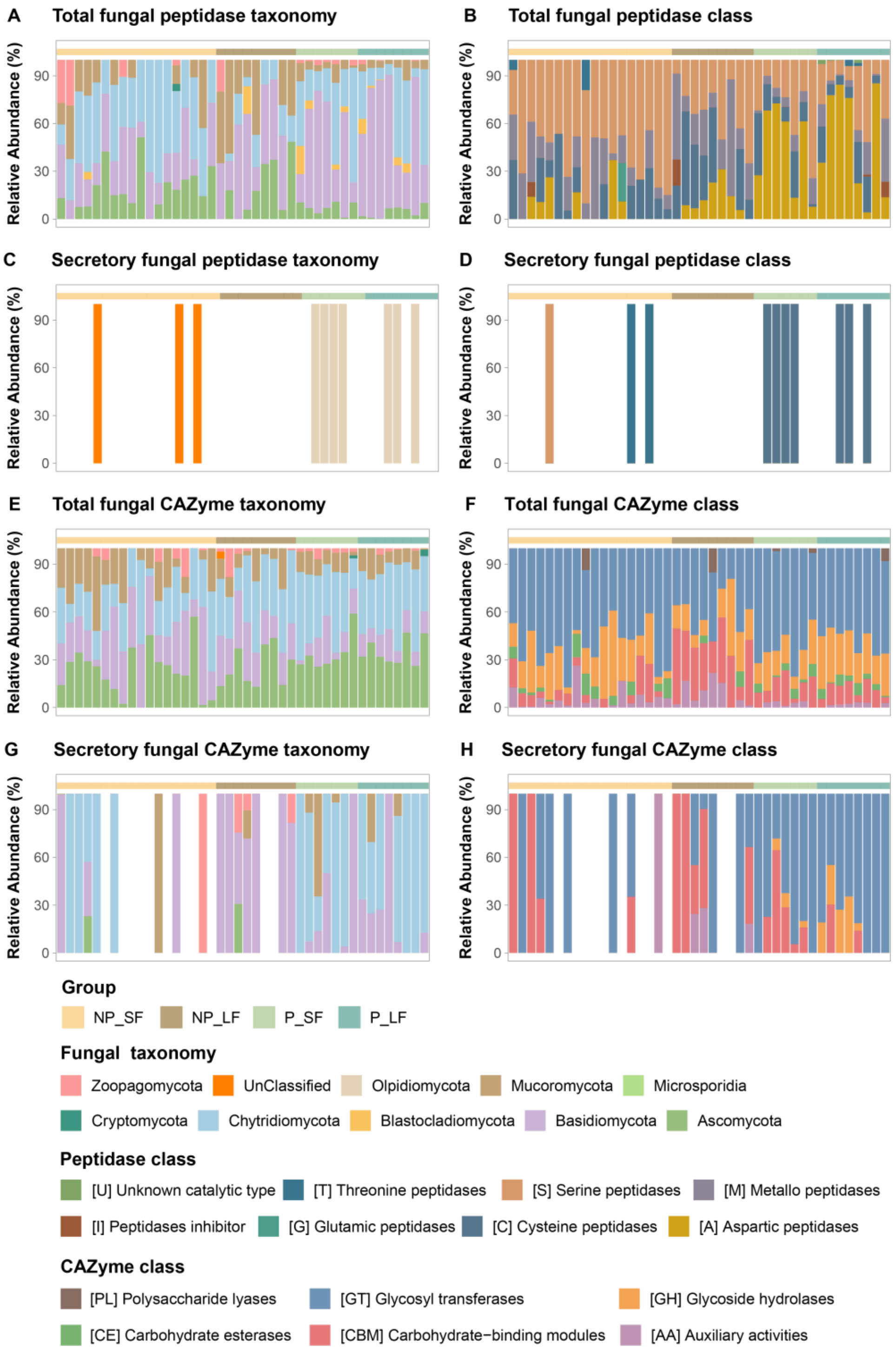

Fig. S3 Stacked bar plots depict the proportion of major phyla in fungal communities and functional classification of genes encoding total and secretory fungal peptidases and CAZymes in the small (0.2-3 μm, SF) and large (> 3 μm, LF) size fractions, as well as between non-polar (NP) and polar (P) oceans. Taxonomic affiliation and functional composition of genes encoding total and secretory peptidases (**A-D**) and CAZymes (**E-H**). For metagenomic analyses, a total of 42 genomic DNA samples were used: NP_SF (n = 18), NP_LF (n = 9), P_SF (n = 7), and P_LF (n = 8). For metatranscriptomic analyses, 53 RNA

samples were analyzed: NP_SF (n = 18), NP_LF (n = 16), P_SF (n = 9), and P_LF (n = 10). Missing columns in the barplot represent samples with no fungal reads detected.

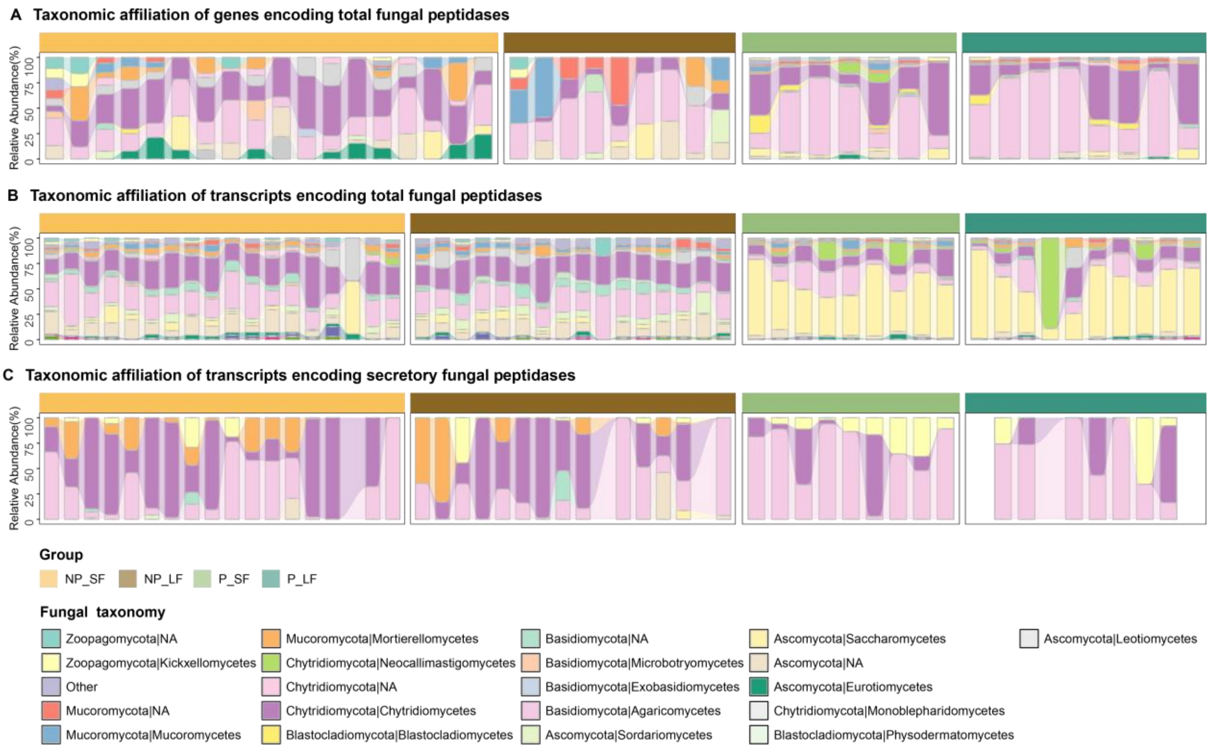

Fig. S4 Stacked bar plots depict the proportion of major classes in fungal communities of genes and transcripts encoding total and secretory fungal peptidases between in the small (0.2-3 μm, SF) and large (> 3 μm, LF), as well as between non-polar (NP) and polar (P) oceans. Taxonomic affiliation of total peptidases at the gene level (**A**) and of total and secretory peptidases at the transcript level (**B-C**). Taxonomic affiliation of secretory peptidases at the gene level was not plotted, as few secretory fungal reads were identified in the samples, as also shown in Fig. S3C. For detailed taxonomic information, please refer to the online version. The sample size was definded in Fig. S3. Missing columns in the barplot represent samples with no fungal reads detected.

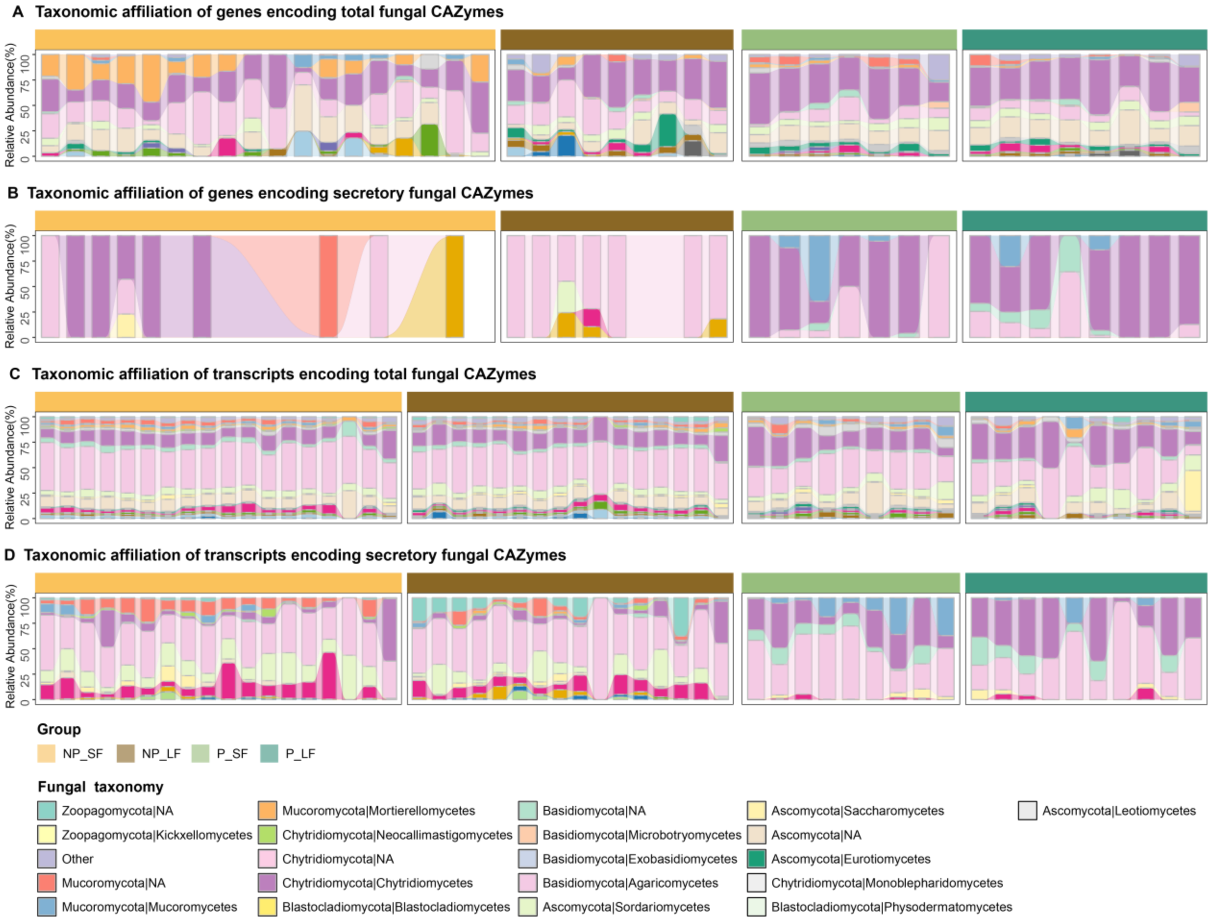

Fig. S5 Stacked bar plots depict the proportion of major classes in fungal communities of genes and transcripts encoding total and secretory fungal CAZymes between in the small (0.2-3 μm, SF) and large (> 3 μm, LF), as well as between non-polar (NP) and polar (P) oceans. Taxonomic affiliation of total and secretory CAZymes at the gene level (A-B) and the transcript level (C-D). For detailed taxonomic information, please refer to the online version. The sample size was definded in Fig. S3. Missing columns in the barplot represent samples with no fungal reads detected.

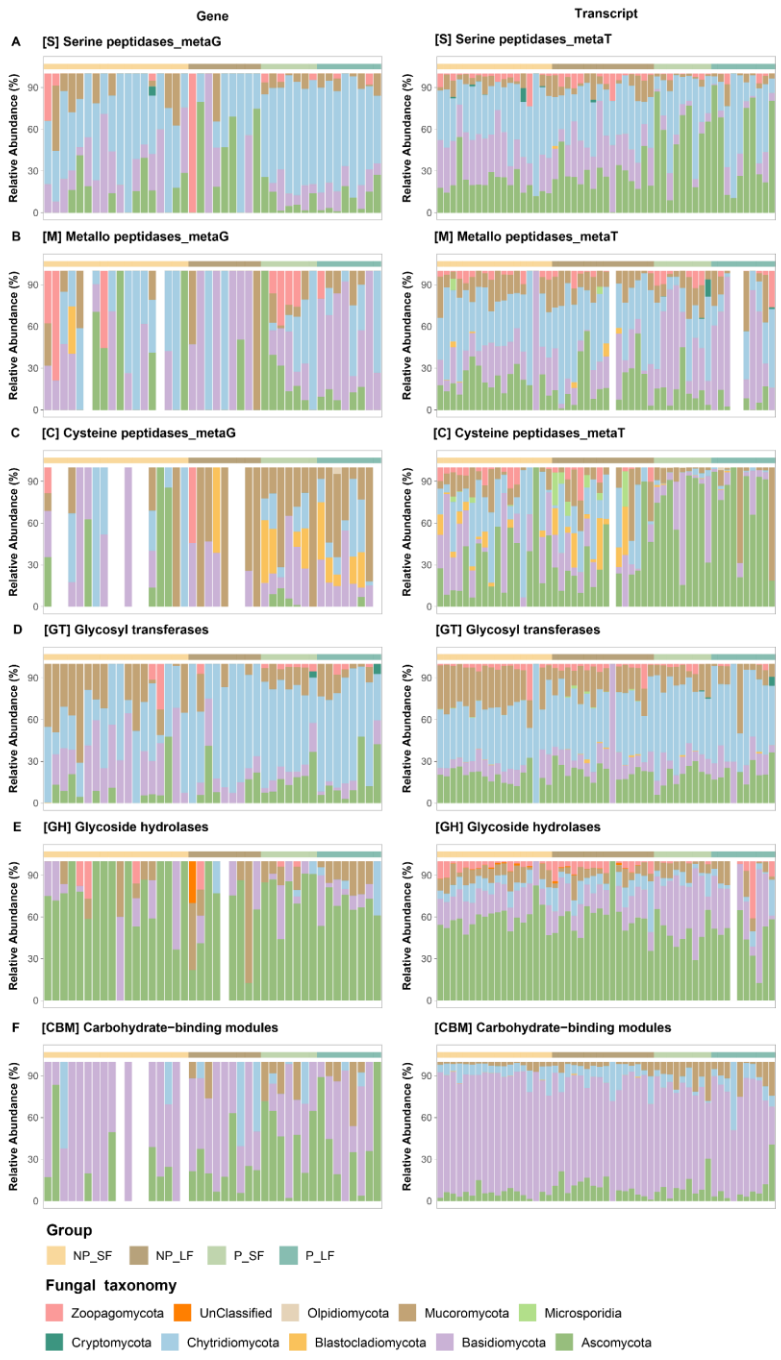

Fig. S6 The contribution of fungal phyla to the expression potential and activity of major peptidase and CAZyme families was analyzed in the small (0.2–3 µm, SF) and large (> 3 µm, LF) size fractions, as well as in non-polar and polar oceans. Fungal genes and transcripts were taxonomically assigned to serine peptidases (**A**), metallo peptidases (**B**), cysteine peptidases (**C**), glycosyl transferases (GTs, **D**), glycoside hydrolases (GHs, **E**), and carbohydrate-binding modules (CBMs, **F**). The sample size was definded in Fig. S3. Missing columns in the barplot represent samples with no fungal reads detected.

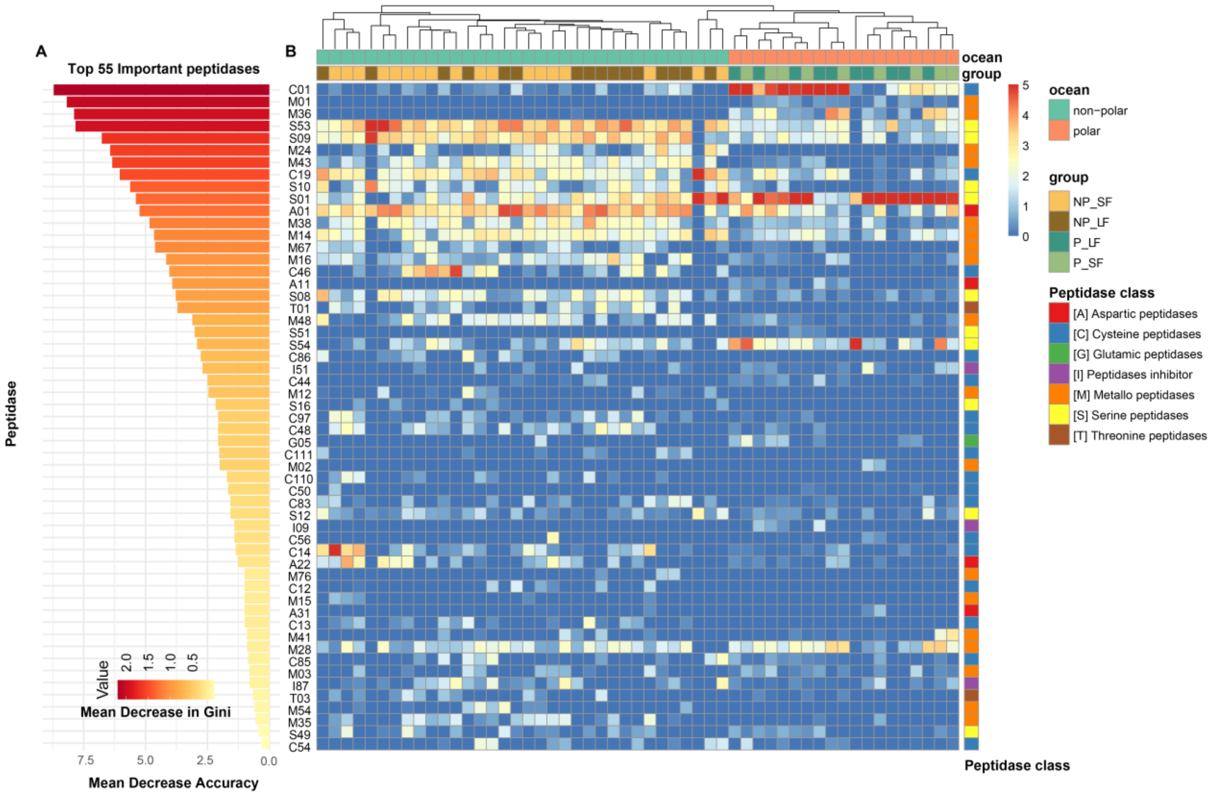

Fig. S7 The transcript expression of top 55 important fungal peptidase identified using random forest classification classes. Top 55 fungal peptidase families ranked by importance (A) and their abundance profiles (B).The relative abundance of peptidase classes was $log_2(n+1)$ transformed. These 55 fungal peptidase classes were identified as positively influencing the accuracy of the random forest result, with feature types distinguishing between non-polar and polar oceans. The accuracy metric measures the decrease in model performance when each variable is excluded, while the mean decrease in the Gini coefficient reflects the contribution of each variable to the homogeneity of nodes and leaves in the random forest results. 53 RNA samples were analyzed: NP_SF (n = 18), NP_LF (n = 16), P_SF (n = 9), and P_LF (n = 10). The complete random forest results can be found in the Supplementary Data 6.

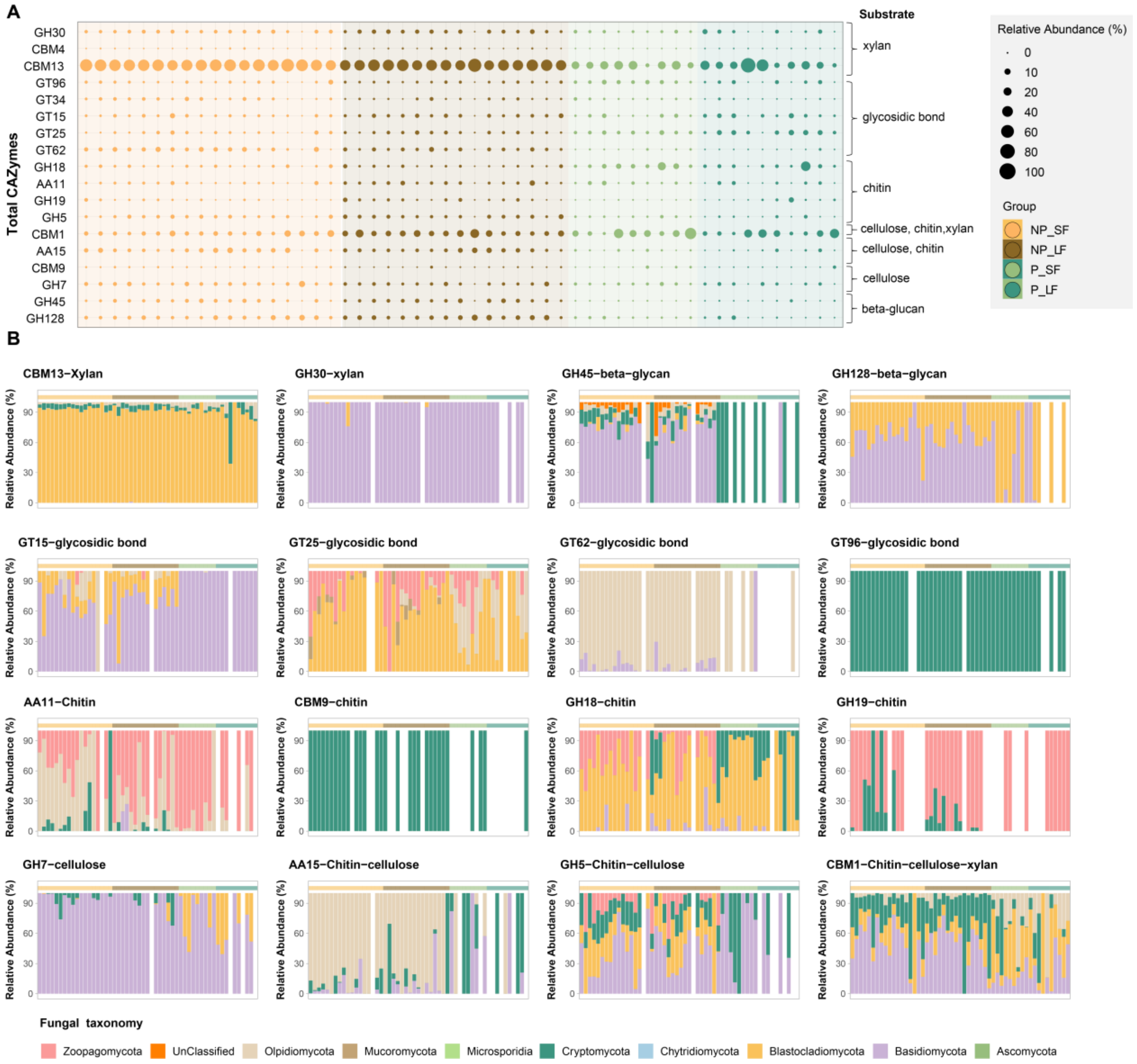

Fig. S8 Relative abundances of CAZyme families in total transcripts with secretion activity (A) and fungal contributions to these substrate-specific CAZymes (B). 53 RNA samples were analyzed: NP_SF (n = 18), NP_LF (n = 16), P_SF (n = 9), and P_LF (n = 10). Missing columns in the bar plots represent samples with no fungal reads detected.

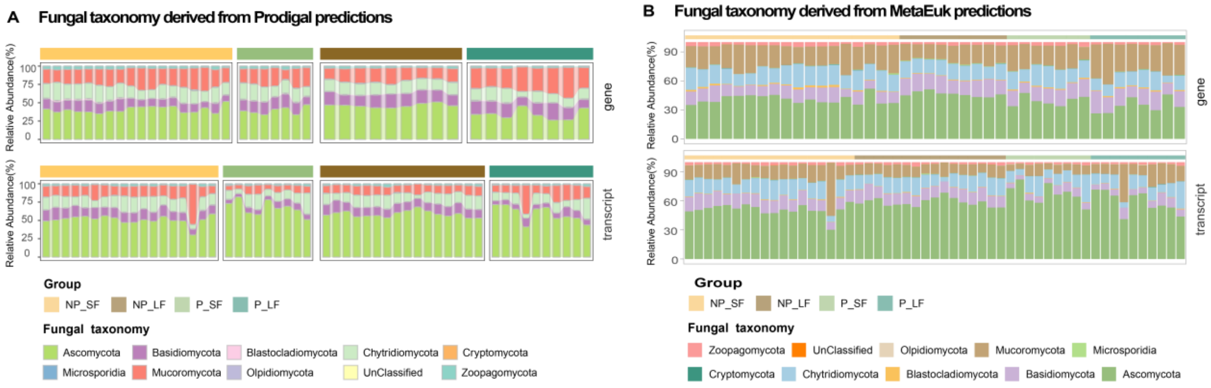

Fig. S9 Taxonomic assignment comparisons of total fungal community composition in the metagenome and metatranscriptome based on Prodigal (A) and Metaeuk (B) putative gene prediction methods. The sample size was definded in Fig. S3.

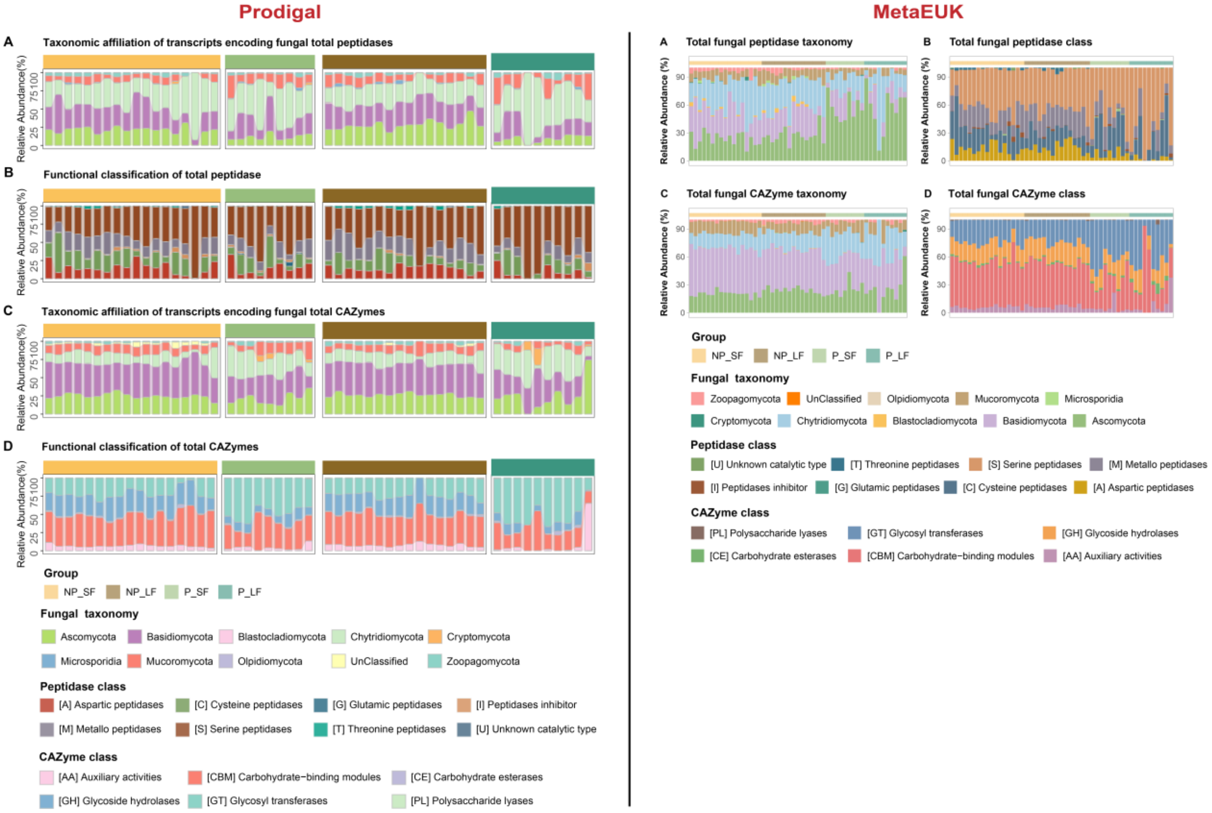

Fig. S10 Comparative analysis of functional annotations using Prodigal (left panel) and MetaEuk (right panel) for peptidase and CAZyme transcript predictions. The sample size was definded in Fig. S3.

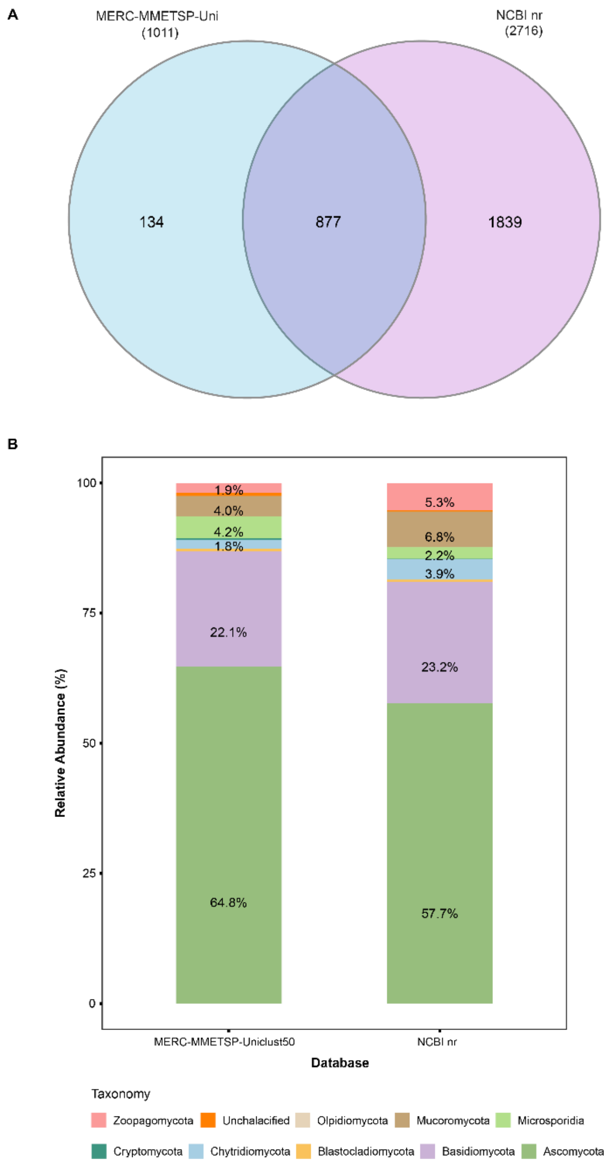

Fig. S11 Overlap and taxonomic consistency between the MERC-MMETSP-Uniclust50 and NCBI nr databases. (A) Majority (86.7%) of taxa identified by the MERC-MMETSP-Uniclust50 database are also present in the NCBI nr database. (B) Taxonomic classification at the phylum level shows high consistency between the two databases.
