# Supplementary material for: Organic matter degradation by oceanic fungi differs between polar and non-polar waters
Source: Nat Commun. 2025 Aug 15;16:7589. doi: 10.1038/s41467-025-63047-4 (PMC12356891; doi:10.1038/s41467-025-63047-4)
Supplement: Supplementary file 2 — Description of Additional Supplementary Files [file 41467_2025_63047_MOESM2_ESM.pdf]

Description of Additional Supplementary Data

Supplementary Data 1: Sample information and environmental parameters

Supplementary Data 2: Sequencing statistics of 42 metagenomic libraries.

Supplementary Data 3: Sequencing statistics of 53 metatranscriptomic libraries.

Supplementary Data 4: Occurrence of predicted total and secretory peptidases and CAZymes in the metagenome.

Supplementary Data 5: Occurrence of predicted total and secretory peptidases and CAZymes in the metatranscriptome.

Supplementary Data 6: Random forest prediction of prptidase families.

Supplementary Data 7: Random forest prediction of CAZyme families.
