# Supplementary material for: Organic matter degradation by oceanic fungi differs between polar and non-polar waters
Source: Nat Commun. 2025 Aug 15;16:7589. doi: 10.1038/s41467-025-63047-4 (PMC12356891; doi:10.1038/s41467-025-63047-4)
Supplement: Supplementary file 4 — Reporting Summary [file 41467_2025_63047_MOESM4_ESM.pdf]

nature portfolio

Corresponding author(s): Kangli Guo, Federico Baltar

Last updated by author(s): Jul 18, 2025

Reporting Summary

Nature Portfolio wishes to improve the reproducibility of the work that we publish. This form provides structure for consistency and transparency in reporting. For further information on Nature Portfolio policies, see our Editorial Policies and the Editorial Policy Checklist.

Statistics

For all statistical analyses, confirm that the following items are present in the figure legend, table legend, main text, or Methods section.

n/a	Confirmed	
☐	☒	The exact sample size (*n*) for each experimental group/condition, given as a discrete number and unit of measurement
☐	☒	A statement on whether measurements were taken from distinct samples or whether the same sample was measured repeatedly
☒	☐	The statistical test(s) used AND whether they are one- or two-sided<br>*Only common tests should be described solely by name; describe more complex techniques in the Methods section.*
☒	☐	A description of all covariates tested
☒	☐	A description of any assumptions or corrections, such as tests of normality and adjustment for multiple comparisons
☐	☒	A full description of the statistical parameters including central tendency (e.g. means) or other basic estimates (e.g. regression coefficient) AND variation (e.g. standard deviation) or associated estimates of uncertainty (e.g. confidence intervals)
☐	☒	For null hypothesis testing, the test statistic (e.g. *F*, *t*, *r*) with confidence intervals, effect sizes, degrees of freedom and *P* value noted<br>*Give P values as exact values whenever suitable.*
☒	☐	For Bayesian analysis, information on the choice of priors and Markov chain Monte Carlo settings
☒	☐	For hierarchical and complex designs, identification of the appropriate level for tests and full reporting of outcomes
☐	☒	Estimates of effect sizes (e.g. Cohen's *d*, Pearson's *r*), indicating how they were calculated

Our web collection on statistics for biologists contains articles on many of the points above.

Software and code

Policy information about availability of computer code

Data collection	No software was used to collect the data
Data analysis	The open-source code used to analyze the data in this study is described in the Materials and Methods section.

For manuscripts utilizing custom algorithms or software that are central to the research but not yet described in published literature, software must be made available to editors and reviewers. We strongly encourage code deposition in a community repository (e.g. GitHub). See the Nature Portfolio guidelines for submitting code & software for further information.

Data

Policy information about availability of data

All manuscripts must include a data availability statement. This statement should provide the following information, where applicable:

- Accession codes, unique identifiers, or web links for publicly available datasets
- A description of any restrictions on data availability
- For clinical datasets or third party data, please ensure that the statement adheres to our policy

Raw metagenomic reads have been deposited to the National Center for Biotechnology (NCBI) under Bioproject number PRJNA1116066;
Raw metatranscriptomic reads have been deposited to the National Center for Biotechnology (NCBI) under Bioproject number PRJNA1115042.

Research involving human participants, their data, or biological material

Policy information about studies with human participants or human data. See also policy information about sex, gender (identity/presentation), and sexual orientation and race, ethnicity and racism.

Reporting on sex and gender	Not applicable
Reporting on race, ethnicity, or other socially relevant groupings	Not applicable
Population characteristics	Not applicable
Recruitment	Not applicable
Ethics oversight	Not applicable

Note that full information on the approval of the study protocol must also be provided in the manuscript.

Field-specific reporting

Please select the one below that is the best fit for your research. If you are not sure, read the appropriate sections before making your selection.

☐ Life sciences ☐ Behavioural & social sciences ☒ Ecological, evolutionary & environmental sciences

For a reference copy of the document with all sections, see nature.com/documents/nr-reporting-summary-flat.pdf

Ecological, evolutionary & environmental sciences study design

All studies must disclose on these points even when the disclosure is negative.

Study description	We utilized metagenomic and metatranscriptomic approaches to uncover fungal functional diversity, biogeography, activity, and their role as metabolic degraders of organic matter in the non-polar and polar ocean. In total, 25 stations and 53 samples covering different sampling depths in both surface (5-20 m) and DCM (25-100 m) layers and size fractions, encompassing both the free-living and particle-attached lifestyle of fungal communities, were collected.
Research sample	Marine samples were collected from surface and deep chlorophyll maximum layers. We conducted high-resolution spatial analyses spanning from subtropical to polar regions in the open Atlantic Ocean and Southern Ocean, differentiating between free-living (0.2 - 3 µm) and particle-attached (> 3 µm) communities. In total, forty-two DNA and fifty-three RNA samples were subjected to sequencing.
Sampling strategy	Details of the sampling strategy can be found in the manuscript in the Materials and Methods section. Briefly, the samples were collected during oceanographic research cruises ANTOM-I and ANTOM-II in 2020 and 2021. The stations were selected to provide a broad representation of oceanic ecosystems in the Atlantic (non-polar) and Southern (polar) Oceans spanning a broad longitudinal gradient across the subtropical to polar oceans. Thirty-one to 106 liters of seawater from the surface and deep chlorophyll maximum (DCM) layers were sequentially filtered with a McLane in-situ pump through >3 µm (hereafter, communities referred to as particle-attached "PA" lifestyle) and 0.2 µm (hereafter, communities referred to as free-living "FL" lifestyle) polycarbonate filters (Millipore). The filters were then immediately stored at −70°C until DNA and RNA extraction.
Data collection	The samples were collected during the oceanographic research cruises ANTOM-I (15 December - 15. January, 2020/2021) and ANTOM-II (January 23 - February 6, 2022).
Timing and spatial scale	All water samples were collected in a single sampling event during oceanographic research cruises ANTOM-I and ANTOM-II in 2020 and 2022.
Data exclusions	No data were excluded from the analyses.
Reproducibility	Samples for sequencing were collected from diverse marine environments.
Randomization	The work was performed on independent stations. Randomization does not apply to field collection as sample sites were predetermined based on oceanographic features.
Blinding	Blinding was not possible as all samples analyzed were associated with a specific site, depth, and fraction size. Clustering approaches allowed for structural patterns in the data to be computationally identified regardless of assigned category.

Did the study involve field work? ☒ Yes ☐ No

Field work, collection and transport

Field conditions	The relevant parameters of field conditions can be found in Supplemental Table S1.
Location	The locations of the sampling sites are detailed in Supplemental Data S1. The stations were selected to provide a broad representation of oceanic ecosystems in the Atlantic (non-polar) and Southern (polar) Oceans spanning a broad longitudinal gradient across the subtropical to polar oceans.
Access & import/export	The oceanographic cruises will be part of the international ANTOM (Transport and biogeochemistry of emerging pollutants and ANThropogenic Organic Matter in the Southern Ocean) project, which is an interdisciplinary experiment in the Atlantic and in the Southern Ocean.
Disturbance	No disturbance was caused by oceanographic sample collection.

Reporting for specific materials, systems and methods

We require information from authors about some types of materials, experimental systems and methods used in many studies. Here, indicate whether each material, system or method listed is relevant to your study. If you are not sure if a list item applies to your research, read the appropriate section before selecting a response.

Materials & experimental systems

n/a	Involved in the study
☒	☐ Antibodies
☒	☐ Eukaryotic cell lines
☒	☐ Palaeontology and archaeology
☒	☐ Animals and other organisms
☒	☐ Clinical data
☒	☐ Dual use research of concern
☒	☐ Plants

Methods

n/a	Involved in the study
☒	☐ ChIP-seq
☒	☐ Flow cytometry
☒	☐ MRI-based neuroimaging

Plants

Seed stocks	Not applicable
Novel plant genotypes	Not applicable
Authentication	Not applicable
